# Tea Ingredients Have Anti-coronavirus Disease 2019 (COVID-19) Targets Based on Bioinformatics Analyses and Pharmacological Effects on LPS-Stimulated Macrophages

**DOI:** 10.3389/fnut.2022.875765

**Published:** 2022-05-20

**Authors:** Lei Wang, Qing Tao, Zhiguo Wang, Jianfeng Shi, Wei Yan, Li Zhang, Yaoxiang Sun, Xiaoming Yao

**Affiliations:** ^1^Department of Clinical Laboratory, Jiangsu Province Hospital on Integration of Chinese and Western Medicine, Jiangsu Province Academy of Traditional Chinese Medicine, Nanjing, China; ^2^Center for Translational Medicine and Jiangsu Key Laboratory of Molecular Medicine, Department of Basic Medicine, Medical School of Nanjing University, Nanjing, China; ^3^Department of Clinical Laboratory, The Affiliated Yixing Hospital of Jiangsu University, Yixing, China

**Keywords:** COVID-19, molecular docking, network pharmacology, tea ingredients, macrophage, key targets

## Abstract

Coronavirus disease 2019 (COVID-19) is a contagious disease caused by severe acute respiratory syndrome coronavirus 2 (SARS-CoV-2) that caused millions of deaths and lacks treatment. Although several studies have focused on the major component of green tea, epigallocatechin 3-gallate (EGCG), which is efficient in preventing COVID-19, systemic analyses of the anti-COVID-19 potential of green tea remain insufficient. Here, we co-analyzed the target genes of tea ingredients and COVID-19 signature genes and found that epigallocatechin 3-acetalbehyde was capable of reversing the major molecular processes of COVID-19 (MAPK and NF-κB activation). These findings were further supported by Western blotting (WB), immunofluorescence, and quantitative polymerase chain reaction (qPCR) in LPS-stimulated macrophages. Moreover, using molecular docking analysis, we identified three tea ingredients ((-)-catechin gallate, D-(+)-cellobiose, and EGCG) that may interact with the vital SARS-CoV-2 protein, 5R84, compared with the qualified 5R84 ligand WGS. Thus, our results indicated that tea ingredients have the potential to treat COVID-19 by suppressing the COVID-19 signature genes and interacting with the vital SARS-CoV-2 protein.

## Introduction

Coronavirus disease 2019 (COVID-19) is a disease with main manifestations involving the lungs and is caused by severe acute respiratory syndrome coronavirus 2 (SARS-CoV-2) (1). SARS-CoV-2 is rapidly spreading around the world, and the number of confirmed cases and infection-related deaths are increasing every day (2). The severity of COVID-19 is associated with increased inflammatory and chemokine factors; these factors also predict COVID-19 mortality (3). Although the pathogenesis of COVID-19 is not fully understood, the virus and host immune system play key roles in its development (4).

From Delta to Omicron, the new coronavirus is constantly mutating, the global epidemic is at a high level, and the number of infections continues to increase (5). While COVID-19 vaccines can greatly prevent the spread of the virus, they cannot treat patients infected with the virus (6). To treat patients with new coronavirus pneumonia, scientists have made considerable efforts in drug research and development; however, to date, there are still very few drugs that can treat COVID-19 (7). Although some neutralizing antibodies and small molecule inhibitors are being developed, there is uncertainty about their safety and efficacy (8). Therefore, we urgently need to explore new strategies to treat COVID-19.

Tea is popular all over the world as a food drink; in fact, tea has been used as an herbal medicine to prevent and treat various diseases (9). Tea and its characteristic polyphenols—catechins—have been shown to be active in preventing obesity, diabetes, cardiovascular disease, cancer, and other diseases (10–12). Tea ingredients have also been shown to have anti-viral activity as well as protective activity against diseases caused by oxidative stress and inflammation; many of these ingredients may help alleviate and treat COVID-19 (13, 14). Although several studies have focused on epigallocatechin gallate (EGCG), the major component of green tea, which has been shown to be effective in preventing COVID-19 (15), we focused on systematic research of the therapeutic potential of tea components for COVID-19, including inhibition of COVID-19 signature gene transcription and direct interactions with specific COVID-19 proteins. Systematic research about tea and COVID-19 currently remains insufficient. Systematic analyses of the anti-COVID-19 potential of green tea and other teas remains insufficient. In this study, we mainly used bioinformatics and computational network-based pharmacology to explore and determine the efficacy and possible therapeutic mechanisms of tea for the treatment of COVID-19 to reveal the potential uses of tea in the treatment of COVID-19. Using a network pharmacology strategy, we report the pharmacological targets and molecular pathways of tea ingredients. Therefore, in this bioinformatics report, we aimed to reveal the component-target-pathway network and pharmacological mechanisms of tea ingredients in the prevention and treatment of COVID-19.

## Materials and Methods

### Identification of the Target Genes of Tea in the Treatment of COVID-19

Using effective tools such as Traditional Chinese Medicine Systems Pharmacology (TCMSP), Swiss Target Prediction, and SuperPred, the target genes of tea were screened from existing databases (16, 17). Other genes related to the occurrence of COVID-19 were obtained using the DisGeNET and GeneCards databases (18). In addition, these putative tea and COVID-19 genes were mapped using the UniProt tool prior to correction. After functional enrichment analysis using FunRich software, all anti-COVID-19 targets of tea were screened and identified.

### Protein-Protein Interaction (PPI) of Candidate Genes

After obtaining the targets of tea and COVID-19, the STRING database was used to further determine and construct a functional protein association network according to a specific algorithm (19, 20). In addition, based on the merged targets of tea and COVID-19, a protein-protein interaction (PPI) network was constructed using Cytoscape software (21, 22). Therefore, the key targets of tea in the treatment of COVID-19 were revealed, visualized, and determined with the topology parameters of the network analyzer tool (23, 24).

### Enrichment Analyses and Kyoto Encyclopedia of Genes and Genomes (KEGG) Pathway of Intersection Targets

R language packages, such as ClusterProfiler, org.Hs.eg.Db, ReactomePA, and GOplot (3.6.1), have been used for enrichment analysis and visualization of the biological processes and Kyoto Encyclopedia of Genes and Genomes (KEGG) pathways of intersection targets (25). In addition, gene annotation information from org.Hs.eg.Db (26, 27), a *p*-value cutoff = 0.05, and a *q*-value cutoff = 0.05 were used for enrichment before plotting the corresponding bubble chart, histogram, and Circos circle chart.

### Molecular Docking Analysis

To screen and identify key targets for tea-based molecular docking assays, a chemical-protein binding approach was used (28, 29). After searching for a specific protein through the PDB database, the 5R84 protein was selected for docking with the tea compound. The three-dimensional structure of tea was drawn using ChemBio3D Draw in Chem Bio Office 2010 software before docking the molecular structure with AutoDock Vina software (30). The plausibility of the docking parameter settings was assessed by the root-mean-square deviation (RMSD) of the ligand molecules. An RMSD ≤ 4 Å is the threshold for ligand molecular conformation.

### Cell Culture

Murine macrophage RAW 264.7 cells were acquired from the Type Culture Collection of the Chinese Academy of Sciences (Shanghai, China). Cells were grown at 37°C under 5% CO_2_ in Dulbecco's modified Eagle's medium (DMEM) supplemented with 10% (v/v) fetal bovine serum (FBS) and 1% penicillin streptomycin (Gibco, USA) in humidified incubators (Thermo, USA). Lipopolysaccharide (LPS, Escherichia coli 055: B5) and EGCG were purchased from Sigma Chemical Co. (St. Louis, USA). RAW 264.7 cells were treated with LPS, LPS+EGCG, or EGCG (for the concentrations of LPS and EGCG see the figure legend) for 24 h. For viability testing, the cells were starved for 24 h without serum before challenge and seeded at a density of 1 × 10^5^ cells/mL in 96-well plates with four replications, and cell viability was analyzed with a CCK-8 cell counting kit (Vazyme, China).

### Quantitative Real-Time Polymerase Chain Reaction (QPCR)

The total RNA was isolated from cells using an RNA extraction kit (Vazyme, China). First-strand complementary DNA (cDNA) was synthesized using an iScript cDNA Synthesis Kit (Vazyme, China). Quantitative PCR was performed with SYBR green PCR Master Mix (Vazyme, China) using a ViiA 7 Real-Time PCR System (Applied Biosystems, CA). The primers are detailed in Table 1. The following cycle parameters were used: 55°C for 2 min, 95°C for 10 min, and 40 cycles of 95°C for 30 s and 60°C for 30 s. The relative expressions of the target genes against that of the reference gene, glyceraldehyde-3-phosphate dehydrogenase (GAPDH), were calculated using the 2^−Δ*ΔCT*^ method. Cell samples were evaluated in triplicate, and every experiment was performed at least three times. The transcription levels of inducible nitric oxide synthase (iNOS), tumor necrosis factor alpha (TNF-α), interleukin (IL)-1β, IL-6, Arg-1, and GAPDH were determined.

**Table 1:** Primers used for real-time quantitative PCR analysis.

**Gene**	**Forward primer**	**Reverse primer**
*iNOS*	ACTCAGCCAAGCCCTCACCTAC	TCCAATCTCTGCCTATCCGTCTCG
*TNF-α*	CAGGCGGTGCCTATGTCTC	CGATCACCCCGAAGTTCAGTAG
*IL-1β*	GCAACTGTTCCTGAACTCAACT	ATCTTTTGGGGTCCGTCAACT
*IL-6*	CCAAGAGGTGAGTGCTTCCC	CTGTTGTTCAGACTCTCTCCCT
*Arg-1*	CATATCTGCCAAAGACATCGTG	GACATCAAAGCTCAGGTGAATC
*GAPDH*	CATCCCAGAGCTGAACG	CTGGTCCTCAGTGTAGCC

### Protein Extraction and Western Blotting (WB)

Total cellular proteins were extracted using radioimmunoprecipitation assay buffer containing 1% sodium dodecyl sulfate (SDS); 40 mg of total lysate was separated by 10% SDS-polyacrylamide gel electrophoresis (SDS-PAGE) gel and transferred to a polyvinylidene fluoride membrane, blocked with 5% bovine serum albumin in tris-buffered saline for 90 min, and then incubated with the appropriate primary antibody overnight at 4°C. Membranes were incubated with secondary antibody for 90 min at room temperature after washing and then visualized using ECL Plus Western Blot Detection Reagent (Millipore, USA). The protein expression levels of extracellular signal-regulated kinase (ERK), p-ERK, c-Jun amino-terminal kinase (JNK) and p-JNK, and p38 and p-p38 were determined by Western blotting (WB). GAPDH was used as an internal control.

### Enzyme Linked Immunosorbent Assay (ELISA)

According to the manufacturer's instructions, the cell supernatant concentrations of IL-6, TNF-α, and IL-1β were determined using ELISA kits (ExCell Bio, China).

### Immunofluorescence Assay

The expression of phospho-p65 was detected by immunofluorescence assays using a fluorescence microscope. RAW 264.7 cells were cultured directly on glass coverslips in 6-well plates for 24 h. After stimulation with LPS in the presence or absence of EGCG, the cells were fixed with 4% paraformaldehyde in PBS. The membrane was permeabilized by treating the cells for 5 min with 0.1% Triton X-100 in PBS. After a brief washing in PBS, slides were blocked with 5% bovine serum albumin for 1 h and then incubated with rabbit polyclonal anti-human phopho-p65 antibody (dilution, 1:100) overnight at 4°C at room temperature. The next day, the specimens were rinsed with PBS three times. After washing, they were incubated with the secondary antibodies (Alexa Fluor® 594, Thermo Fisher Scientific, CA, USA) for 30 min and counterstained for nuclei with DAPI (Beijing Solarbio Science & Technology, Beijing, China) for 10 min. After a brief washing in PBS, slides were sealed using ProLong® Gold antifade reagent (Molecular Probes® by Life Technologies™, CA, USA). Fluorescence micrographs were acquired with a fluorescence microscope (Nikon ECLIPSE Ti-U, Nikon Co., Japan).

### Data Analysis

Normally distributed data were analyzed using Student's *t*-test (for two-group comparisons) or analysis of variance (for multiple-group comparisons). For non-normally distributed values (as determined by the Kolmogorov–Smirnov test), the Mann–Whitney's rank-sum test was used. All statistical tests were two-sided, and *P* < 0.05 was considered statistically significant. Data are presented as the mean ± standard error of the mean (SEM) and presented using GraphPad Prism 5 software (LaJolla, CA).

## Results

### Identification the Ingredients and Target Genes of Tea

**Figure 1:**
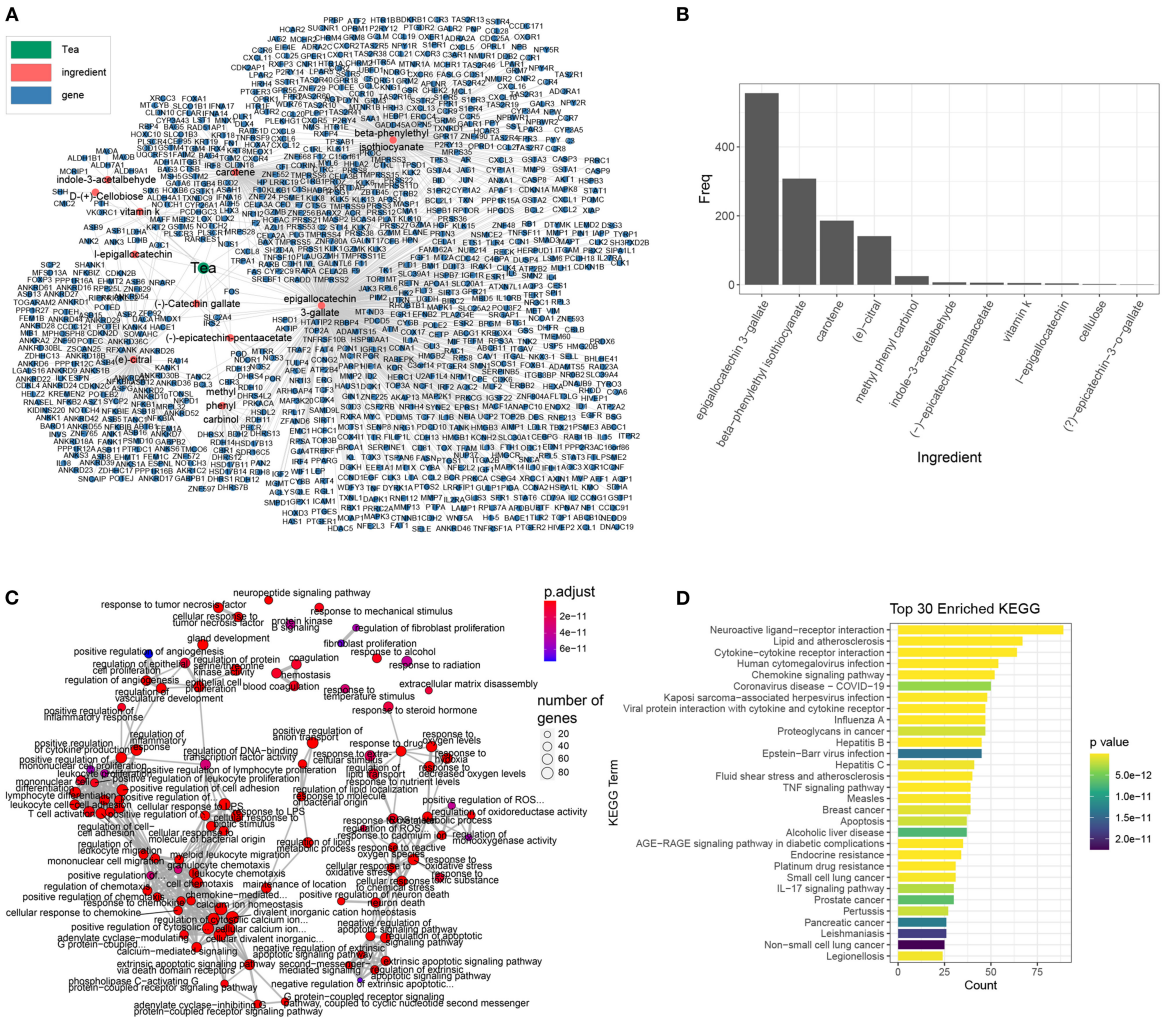
The ingredients and target genes of tea. **(A)** The network of tea ingredients and target genes, the dot color represents the components, while green, red, and blue represents the tea, tea ingredients, and target genes, respectively. **(B)** The target genes bar plot of each tea ingredient. **(C)** The GO enrichment map of tea target genes organized enriched terms into a network with edges connecting overlapping genes and easier to identify hub module. **(D)** The KEGG enrichment bar plot of the top 30 enriched terms, the bar color represents the *P*-value of each term.

We first downloaded the ingredients and target genes of tea from the Traditional Chinese Medicine Integrated Database (TCMID) database (31). Eleven annotated ingredients and 931 target genes were reported, among which EGCG was the major ingredient and targeted 556 genes (Figures 1A,B). According to Gene Ontology (GO) enrichment analysis, the target genes of tea were involved in inflammation and chemokines (positive regulation of cytokine production, positive regulation of leukocyte migration, etc.), coagulation and cell death (neuronal death, extrinsic apoptotic signaling pathways, etc.) (Figure 1C), which were previously reported as the molecular characteristics of COVID-19, indicating that the tea has the potential to have anti-COVID-19 activity. The KEGG enrichment analysis also demonstrated that tea has a strong antiviral activity, with target genes that were functionally enriched in COVID-19 and influenza A, and represses inflammation (Figure 1D).

In addition to EGCG, there are many additional components such as beta-phenylethyl isothiocyanate, carotene, and citral, that also have anti-inflammatory and anti-chemotactic effects for COVID-19. We intersected the targets of other tea ingredients with the signature genes of COVID-19 (Supplementary Figure 1) and found that the targets of EGCG covered the most signature genes of COVID-19. Furthermore, beta-phenylethyl isothiocyanate and carotene also covered some signature genes. The enrichment analysis of the corresponding intersected genes (Supplementary Figure 2) showed that in addition to EGCG, other tea ingredients can repress the corresponding pathological processes involved in COVID-19. For instance, the targets of beta-phenylethyl isothiocyanate are closely related to cell chemotaxis in COVID-19. Citral inhibits inflammation and NK-κB signaling in COVID-19. Finally, cartone is related to coagulation and cytokine secretion. In summary, these results imply that tea can suppress inflammation and prevent coronavirus disease.

### Molecular Characterization of COVID-19 Infection

**Figure 2:**
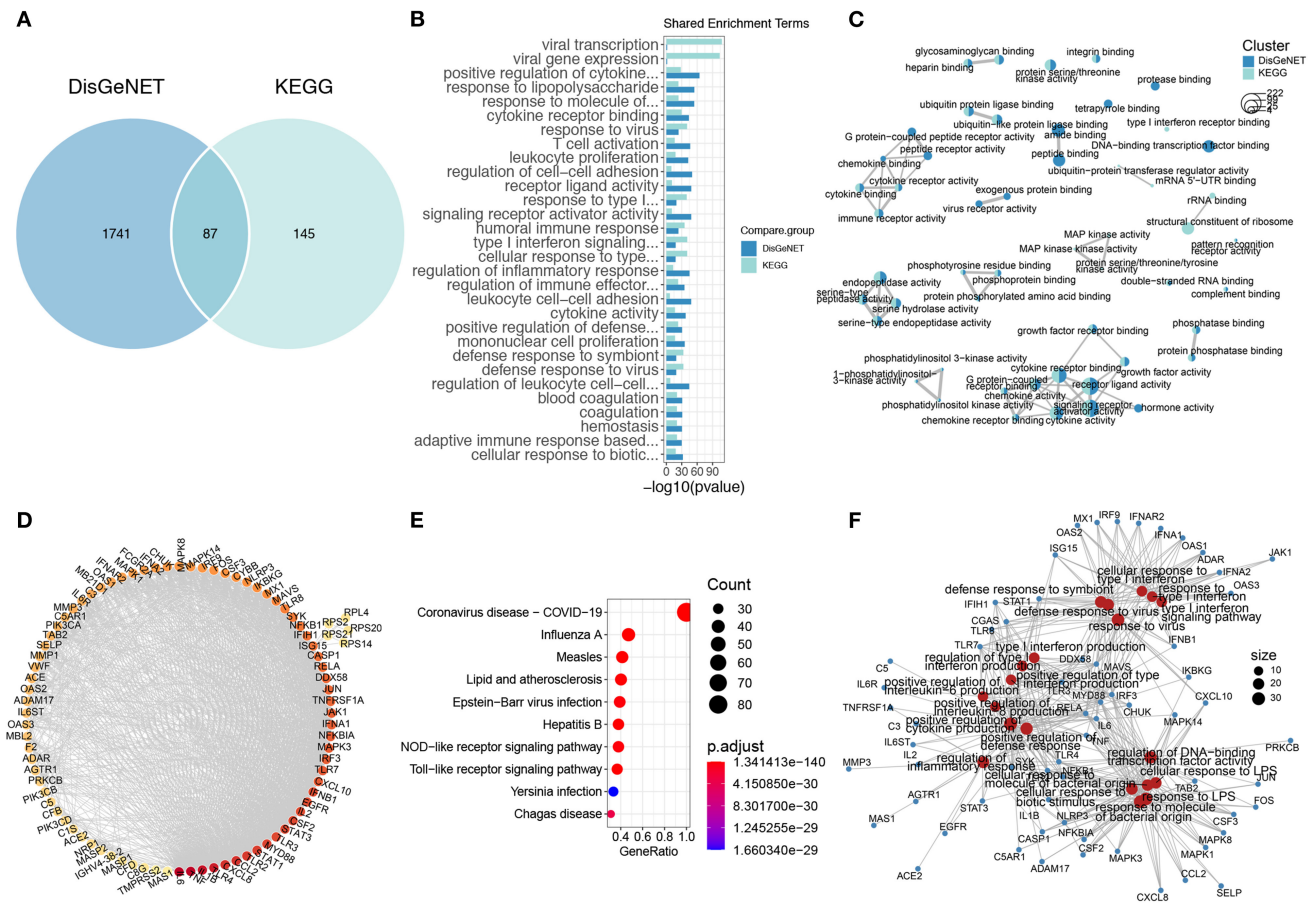
The gene signature of COVID-19. **(A)** The Venn plot of COVID-19 gene signature of DisGeNET and KEGG. **(B)** The shared COVID-19 gene signature GO terms of DisGeNET and KEGG. **(C)** The comparison GO enrichment network of DisGeNET and KEGG, the number of circles in the bottom left corner represents the gene number of each enriched term, the proportion of clusters in the pie chart is determined by the number of genes. **(D)** The PPI of shared genes of DisGeNET and KEGG COVID-19 gene signature, the dot color represents the connectivity of each gene, while from yellow to red represents from low to high. **(E)** The top 10 KEGG enriched terms of shared COVID-19 signature genes. **(F)** The genes and GO enriched terms network of shared COVID-19 signature genes, the red dots represent the GO enriched terms while blue dots are the related genes.

**Figure 3:**
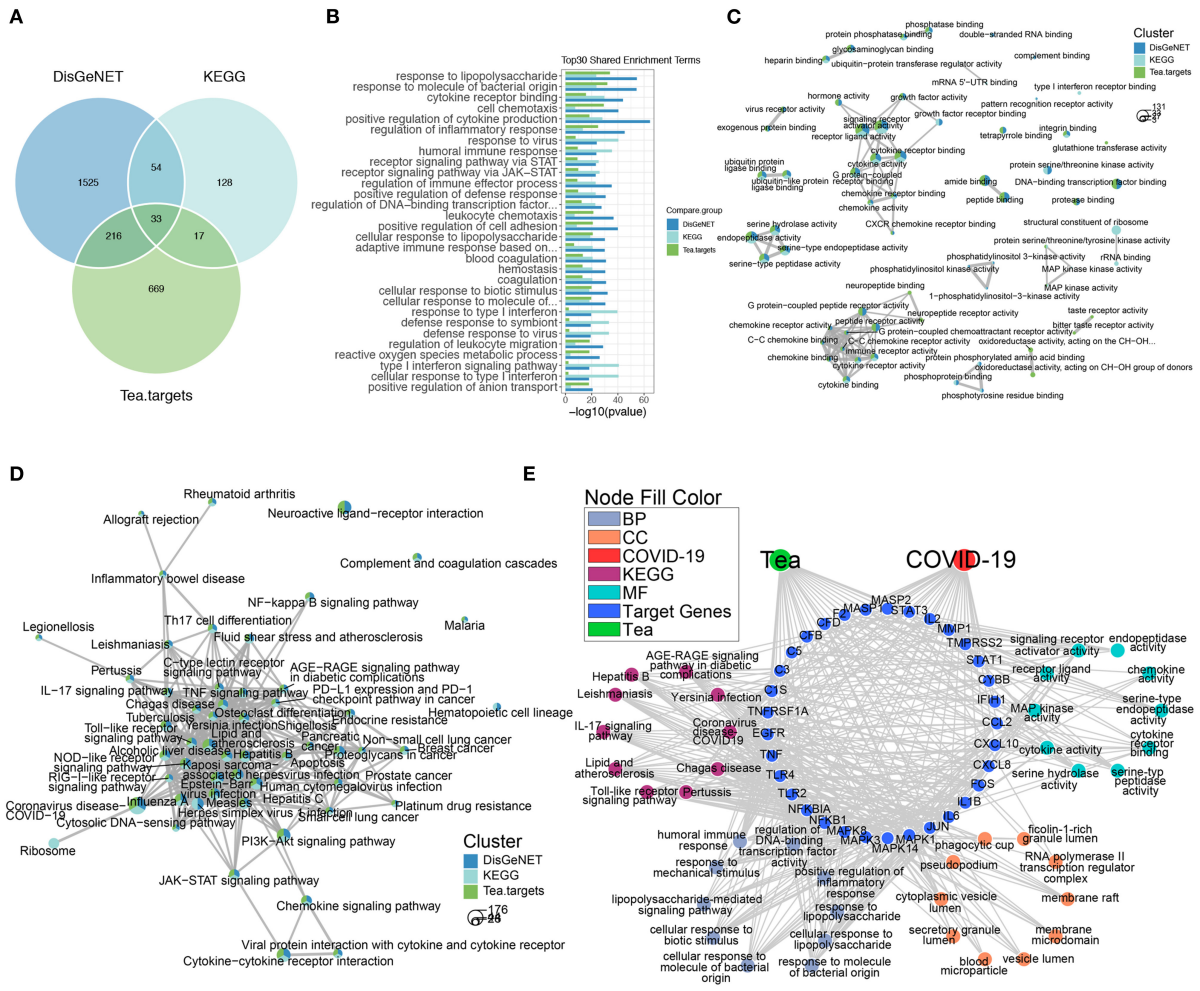
The anti-COVID-19 potential of tea. **(A)** The Venn plot of COVID-19 signature genes and tea target genes. **(B)** The shared GO enriched terms of COVID-19 signature genes and tea target genes. The comparison GO **(C)** and KEGG **(D)** enrichment network of COVID-19 signature genes and tea target genes, the bottom left circles stand for the gene number of each enriched term, the proportion of clusters in the pie chart indicates the number of genes. **(E)** The network of shared COVID-19 signatures genes and tea target genes along with the GO and KEGG enrichment terms.

We next employed the DisGeNET and KEGG databases to characterize the molecular signature of COVID-19 infection. There were 1,288 and 232 COVID-19 signature genes in the DisGeNET and KEGG databases, respectively, among which 87 genes were shared (Figure 2A). These genes were functionally enriched in response to viruses, innate and adaptive immune responses, inflammatory responses, and coagulation (Figure 2B). Notably, the signature genes of COVID-19 infection were also enriched in response to LPS, indicating a similar molecular pattern between COVID-19 infection and sepsis (Figure 2B). The similarity analysis of the enrichment results of the DisGeNET and KEGG COVID-19 genes revealed that cytokine and chemokine activity, endopeptidase activity, and phosphatidylinositol 3-kinase (PI3K) activity were the major processes of COVID-19 infection (Figure 2C). Furthermore, the enrichment of KEGG COVID-19 signature genes showed a MAPK signaling pathway specificity (Figure 3C). As for shared genes of the two databases, the PPI analysis implied that they were highly biologically relevant; among these genes, IL-6, TNF, and IL-1B were relevant to the highest degree (Figure 2D). In addition to cytokines, the Toll-like receptor (TLR2, TLR3, TLR7, and TLR8) and inflammatory signaling pathways (JAK-STAT, NF-κB, and MAPK signaling pathways) were also important components in the PPI network (Figure 2D). The functions of these genes included involvement in the antiviral process (COVID-19, influenza A, etc.) and responses to molecules of bacterial origin and inflammation (Figures 2E,F).

### Identification the Candidate Target Genes of Tea and COVID-19

To further verify the anti-COVID-19 activity of tea ingredients, we co-analyzed the target genes of tea with COVID-19 signature genes. There were 249 and 50 shared targets genes of tea with DisGeNET and KEGG COVID-19 gene signatures, respectively, and 33 shared genes in all conditions (Figure 3A). The shared GO enrichment items were focused on the response to bacteria and viruses, inflammation (cytokines and chemokines), immune responses, and coagulation; these cover the majority of signature genes of COVID-19 that were enriched (Figures 2B, 3B), suggesting that the ingredients of tea might act as anti-COVID-19 components. The comparison of the GO enrichment results also showed that tea could target the critical pathological processes involved in COVID-19 infection including cytokine and chemokine activity, endopeptidase activity, and the MAPK signaling pathway (Figure 3C). The comparison of the KEGG enrichment results also showed a similar pattern that covered the major inflammatory signaling pathways including the JAK-STAT, NF-κB, and MAPK signaling pathways (Figure 3D). Furthermore, the shared 33 genes in all three conditions were functionally involved in inflammation and immune responses, which are similar to the major pathological processes of COVID-19, which involve the Toll-like receptor signaling pathway, IL-17 signaling pathway, and cytokine and chemokine activity (Figure 3E). Notably, the shared 33 genes were similar to the high degree genes in PPI, such as IL-6, TNF, and IL-1β, revealing that they were centrally involved in the pathological status of COVID-19 infection. Thus, the results demonstrated that the target genes of tea covered the critical processes involved in COVID-19 infection and might serve as anti-COVID-19 components.

### Molecular Docking Analysis of Tea Ingredients With the COVID-19 Protein 5R84

**Figure 4:**
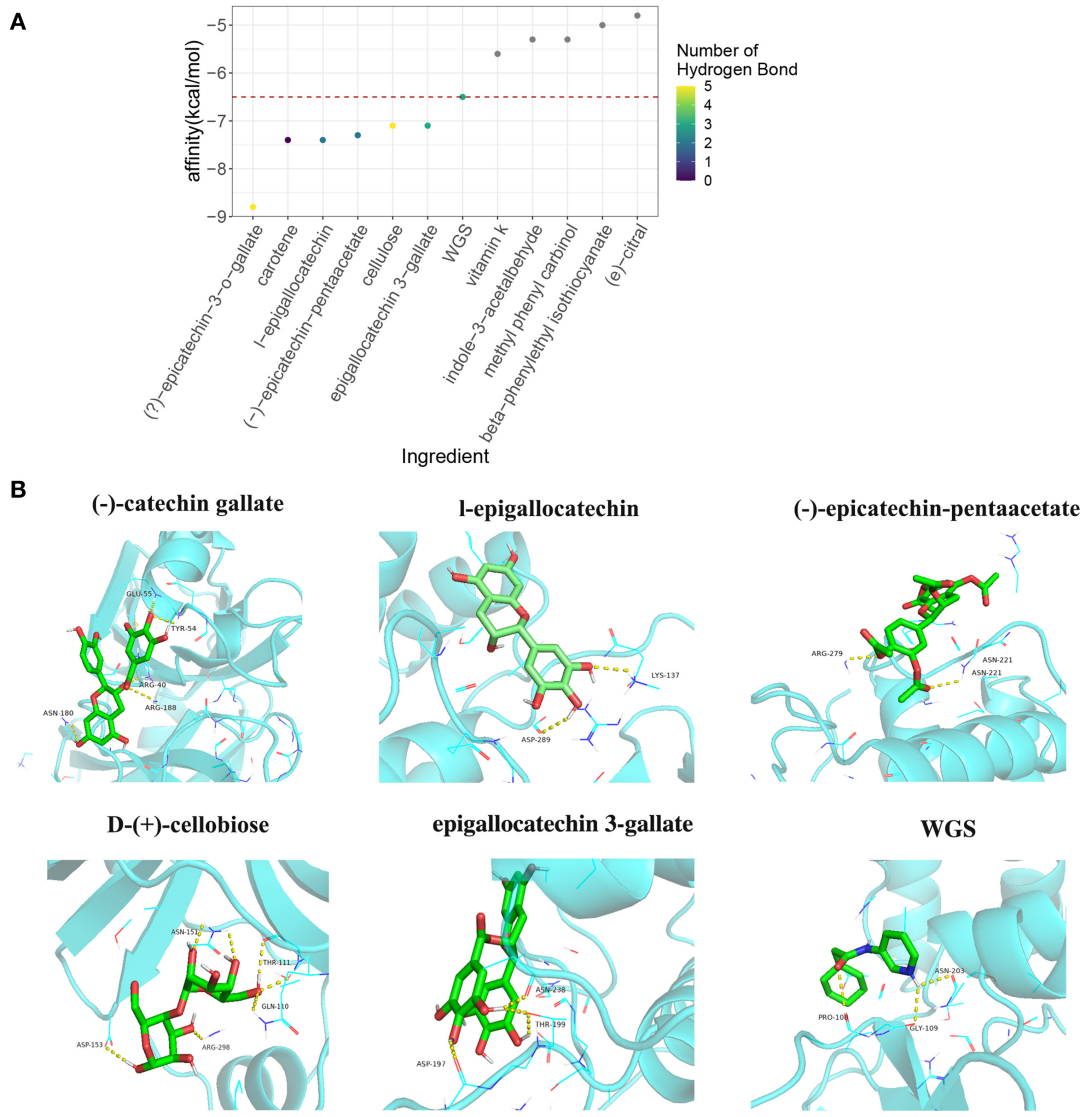
The molecular docking analysis of tea ingredients with COVID-19 5R84 protein. **(A)** The binding-free energy and hydrogen bond numbers of tea ingredients with 5R84 protein with qualified 5R84 ligand WGS as a reference, the red dot horizontal line indicates the binding-free energy of WGS with 5R84. **(B)** The representative interaction of tea ingredients and WGS with 5R84, the yellow dot lines indicate the hydrogen bonds of the specific ligand with 5R84.

Previous studies have reported that small molecules are able to block the COVID-19 virus through interaction with vital virus proteins, such as 5R84 (32). We next examined the interaction of tea ingredients with the COVID-19 protein 5R84 by molecular docking (33). Six of 11 tea ingredients were capable of interacting with 5R84 and had lower free binding energies than the qualified 5R84 ligand WGS; these were (–)-catechin gallate, carotene, l-epigallocatechin, (–)-epicatechin-pentaacetate, D-(+)-cellobiose, and epigallocatechin 3-gallate (Figure 4A). Among these 6 ingredients, (–)-catechin gallate had the lowest binding free energy (−8.8 kcal/mol) and formed 5 hydrogen bonds with the ARG40, TYR54, GLU55, ASN-180, and ARG-188 residues of 5R84 (Figure 4B). The other 5 ingredients shared comparable binding free energies (~-7.26 kcal/mol) and formed 0 to 5 hydrogen bonds with residues (Figure 4B). Notably, although carotene had a low binding free energy, it could not form hydrogen bonds with 5R84, implying that the interaction of carotene with 5R84 was not stable. In summary, we identified three tea ingredients ((–)-catechin gallate, D-(+)-cellobiose, and epigallocatechin 3-gallate) that were sufficient to block COVID-19 by interacting with 5R84 protein.

### Epigallocatechin 3-Gallate (EGCG) Reduced the Secretion of Inflammatory Factors by Inhibiting MAPK/NF-κB Signaling and Regulating Macrophage Polarization *in vitro*

**Figure 5:**
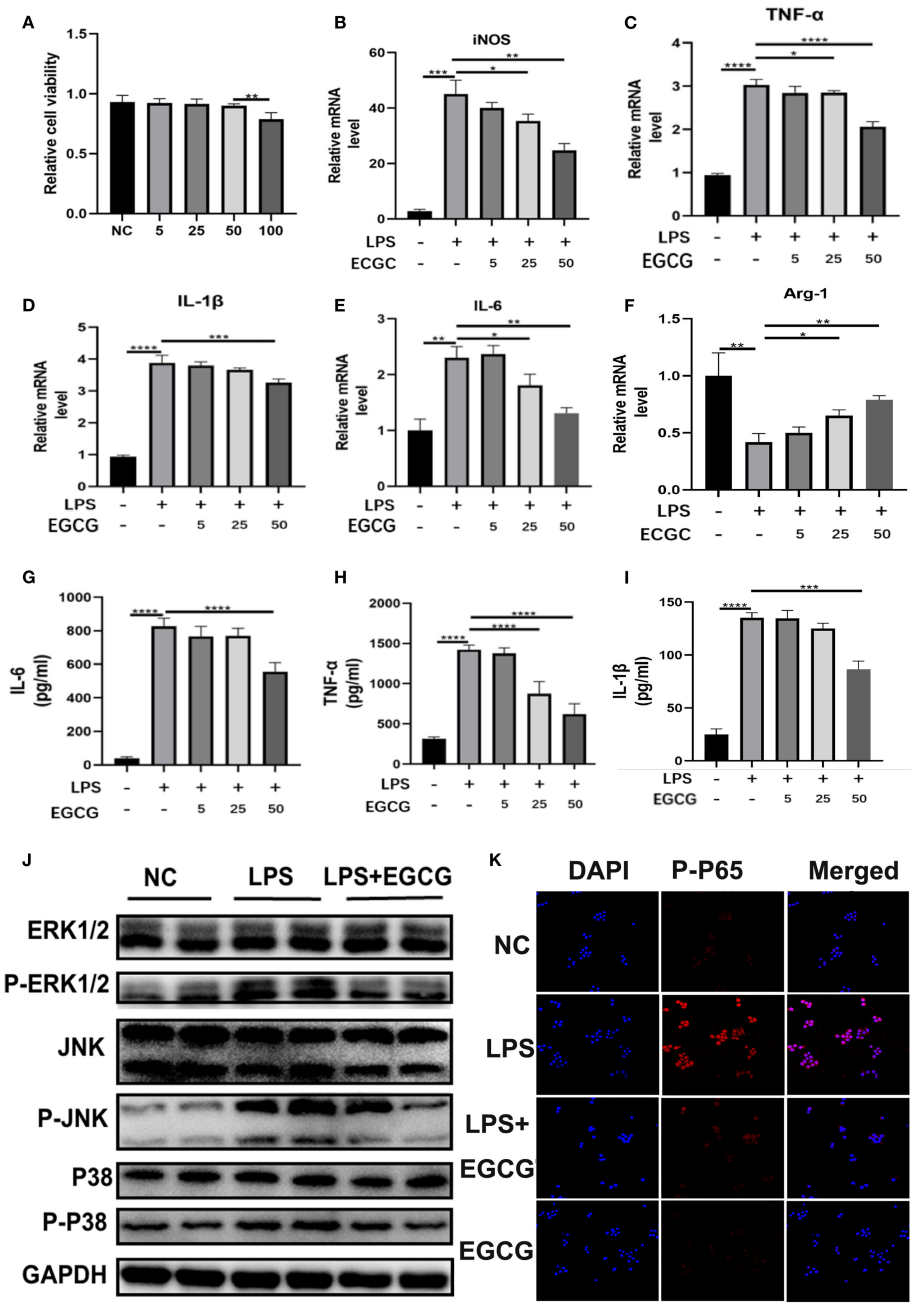
EGCG suppressed secretion of inflammatory factors, macrophage polarization, and MAPK/NF-κB signaling *in vitro*. **(A)** RAW 264.7 cells were incubated with EGCG (50 mM) for 24 h. Cell viability was determined by CCK8 assay (*n* = 5). **(B–F)** The mRNA levels of *iNOS, TNF-*α*, Il-1*β*, IL-6*, and *Arg1* in the RAW 264.7 cells with LPS (100 ng/ml) and EGCG (50 nM) for 24 h were detected by q-PCR (*n* = 3). **(G–I)** The concentrations of IL-6, TNF-α, and IL-1β in RAW 264.7 cell supernatant after LPS and EGCG treatment for 24 h were determined by ELISA kits (*n* = 4). **(J)** The protein levels of ERK1/2, P-ERK1/2, JNK, P-JNK, P38, and p-p38 in the RAW 264.7 cells treated with LPS (100 ng/ml) and EGCG (50 nM) for 24 h were detected by Western blotting. **(K)** The expressions of p-p65 (red) and DAPI (blue) in RAW 264.7 cells were detected by using an immunofluorescence staining assay (scale bar: 50 μm). ^*^P < 0.1, ^**^P < 0.01, ^***^P < 0.001, ****P < 0.0001.

Based on the abovementioned biometric analysis results, it is reasonable to hypothesize that EGCG is involved in inflammation in COVID-19. To ascertain whether EGCG can protect the body from inflammatory injury, we conducted a CCK8 assay. The results revealed that cell viability began to decline when the concentration of EGCG exceeded 50 nM (Figure 5A). Subsequently, we analyzed the effect of EGCG on macrophage polarizations. The LPS (100 ng/mL)-induced mRNA expression of M1 marker genes including *iNOS, TNF-*α*, Il-1*β, and *IL-6* was significantly reduced by EGCG (Figures 5B–E). On the other hand, EGCG showed an increased effect on the level of induction of the M2 marker gene Arg1 stimulated by LPS in RAW264.7 cells (Figure 5F). Then, we collected RAW264.7 cell supernatants after LPS stimulation in a culture system with or without EGCG to measure the secretion of inflammatory factors by ELISA. The results showed that EGCG significantly reduced the production of IL-6, TNF-α, and IL-1β compared with the LPS stimulation group (Figures 5G–I). Moreover, we also detected the inflammatory factor IL-17A secreted by macrophages and the expression of TLR4 and PI3K, which were previously screened (Figure 3E); EGCG significantly suppressed the production of IL-17A and the mRNA levels of TLR4 and PI3K compared with the LPS stimulation group (Supplementary Figure 3). These results indicate that EGCG reduces the secretion of inflammatory factors *in vitro*.

To further explore the mechanism by which EGCG alleviates inflammatory damage to cells, we investigated the inflammation pathway *in vitro*. We measured the activation of the MAPK pathway. As shown in Figure 5J, phosphorylation of p-ERK, p-JNK, and p-p38 in macrophages was significantly increased after LPS challenge; this effect was suppressed by EGCG *in vitro* as determined by WB (Figure 5J). This demonstrates that EGCG could effectively inhibit the MAPK pathway. Furthermore, we investigated the suppressive effect of EGCG treatment on the NF-κB signaling cascade in RAW264.7 macrophages. Our investigations indicated that the phosphorylation of p65 was significantly increased after LPS challenge, and this was suppressed by EGCG (Figure 5K). This finding confirms that EGCG suppressed inflammation by inhibiting MAPK/NF-κB signaling.

## Discussion

Tea is one of the three most consumed beverages in the world and is known as the beverage of the twenty-first century, not only because of the long history of tea culture but also because of its nutritional value and health care functions (34, 35). Studies have shown that tea contains numerous active ingredients, mainly tea polyphenols, tea pigments, tea polysaccharides, γ-aminobutyric acid, tea saponins, alkaloids, vitamins, pyrroloquinoline quinone, pantothenic acid, minerals, and other ingredients (36, 37). Tea polyphenols are the most abundant soluble components in tea, and they are also the most important substances in tea that exert biological effects (35) that can reduce the incidence of cardiovascular disease, decrease blood lipids, decrease body fat formation, and change the intestinal flora ecology (35, 38). Studies have shown that after drinking a cup of tea for half an hour, the antioxidant capacity (ability to fight oxygen free radicals) in the blood increases by 41% to 48% and can last for one and a half hours at a high level (39).

In our work, we first screened the main ingredient of tea, EGCG, in databases, suggesting that EGCG may play an important role in the treatment of COVID-19. EGCG is the main component of green tea polyphenols and is a catechin monomer isolated from tea (40). Studies have shown that EGCG has several functions including significant anti-oxidation, involvement in scavenging free radicals, reduction of inflammation and allergic reactions, anti-mutagenic effects, inhibition of tumor growth, and strong inhibitory effects on dysentery, typhoid fever, *Staphylococcus aureus*, and other bacteria (41–43). EGCG also has the functions of anti-aging, lowering blood lipids, improving low-density lipoprotein, inhibiting the growth of liver fat and cholesterol, preventing atherosclerosis, and enhancing immunity (44–46). In addition, EGCG can inhibit the proliferation of glomerular cell membranes and improve renal function (47). Several studies have reported the potential of EGCG to prevent COVID-19. For instance, EGCG inhibits the angiotensin-converting enzyme 2 (ACE2) receptor (the cellular receptor for SARS-CoV-2) and TMPRSS2, which mediate viral entry into cells, by activating Nrf2 (48, 49). By inhibiting the main protease of SARS-CoV-2, EGCG may inhibit viral reproduction (48). EGCG protects against SARS-CoV-2-induced mitochondrial reactive oxygen species (ROS) (promoting SARS-CoV-2 replication) and ROS bursts caused by neutrophil extracellular traps through its broad antioxidant activity (48, 50). EGCG can potentially inhibit the SARS-CoV-2 life cycle by inhibiting ER-resident GRP78 activity and expression (51, 52). EGCG has also been shown to protect against (1) cytokine storm-related acute lung injury/acute respiratory distress syndrome (48, 53), (2) thrombosis through inhibition of tissue factor and activation of platelets (54), (3) inactivation of redox-sensitive HMGB1-induced sepsis (55), and (4) pulmonary fibrosis by increasing Nrf2 and inhibiting NF-κB (13). However, these activities remain to be further confirmed in animals and humans.

Studies have shown that macrophages play an important role in COVID-19 (56). Cytokine storm syndrome (CSS) refers to the continuous activation and expansion of lymphocytes and macrophages caused by the infection of microorganisms, and a variety of cytokines such as TNF-α, IL-1, IL-6, IL-12, interferon (IFN)-α, IFN-β, IFN-γ, monocyte chemoattractant protein (MCP)-1, and IL-8 are rapidly produced in large quantities (57). CSS is an excessive immune phenomenon of the body to external stimuli and is an important cause of acute respiratory distress syndrome and multiple organ failure (58). Studies have shown that cytokine storms play a key role in the transition to severe and critical illness in most coronavirus-infected patients (59). In addition, one study found that there is a highly pro-inflammatory macrophage microenvironment in the lungs of severely ill patients with the new strain, which the researchers said may help to elucidate the underlying mechanism behind the immune response triggered by the new coronavirus (60). Therefore, we focused on the role of EGCG in regulating changes in macrophage function to improve COVID-19. The inflammatory response in COVID-19 is much more complex than that in LPS-induced RAW264.7 cells, and it is extremely important to distinguish the inflammatory subtypes of different diseases. However, the inflammatory response in COVID-19 still shares some common signatures with the inflammatory response in LPS-induced RAW264.7 cells, among which the most typical are TLR4, NF-κB, and other signaling pathways and their corresponding cytokines (including IL-6, TNF, IL-1β, etc.) (Figure 2D). Through the LPS-stimulated macrophage model, we attempted to demonstrate the possibility of EGCG molecules indirectly inhibiting COVID-19 inflammation.

EGCG has been reported to alleviate acute lung injury, regulate the polarization of macrophages to M2 (61), and inhibit secretion of inflammatory factors, and its protective mechanism may be related to the inhibition of the MAPK and NF-κB signaling pathway (62–64). In addition, EGCG derivatives have anti-inflammatory activity in LPS-stimulated mouse macrophages (65). Furthermore, EGCG-modified collagen membranes have been shown to downregulate the expression of inflammatory factors and promote M2 (CD163 and CD206) macrophages (66). EGCG also stimulates LC3-II production and autophagosome formation and inhibits LPS-induced upregulation and extracellular release of HMGB1 (67). Our results are consistent with those described above; however, the origins of the abovementioned research and our study are different. There is some heterogeneity in the inflammatory responses of different diseases and different states of certain diseases. Starting from the gene signature of COVID-19, we co-analyzed the target genes of each component of tea in an attempt to identify the potential of specific components of tea for the treatment of COVID-19. The results showed that the intersection of COVID-19 signature genes and tea target genes was highly focused on the response to LPS stimulation (Figure 3B). This phenomenon itself is an important discovery. Among the different components of tea, EGCG is obviously an important molecule regulating this process in COVID-19; furthermore, it has the most target genes and is the major active ingredient in tea. We then indirectly verified our findings in LPS-stimulated macrophages *in vitro* to examine the suppression effects of EGCG on the LPS-like responses in COVID-19. Finally, our study is slightly different from the abovementioned literature (61–67) in terms of molecular signaling pathways. Based on the results of the bioinformatics analysis, we focused on the most credible MAPK (ERK1/2-JNK-P38) signaling pathway. In addition, EGCG reduced the secretion of inflammatory factors and regulated macrophage polarization (from M1 to M2) *in vitro*. These cell experiments verified the results of our bioinformatics analysis; namely, the active ingredient of tea, ECGC, can directly act on macrophages in the cytokine storm environment of COVID-19, and inhibit the secretion of inflammatory factors and the activation of the MAPK and NF-κB signaling pathways, improving the prognosis of COVID-19.

Moreover, Douangamath et al. (68) performed a large-scale electrophilic and non-covalent fragment screening of the major proteases of SARS-CoV-2 by combined mass spectrometry and X-ray and found that 5R84 is one of two cysteine viral proteases essential for viral replication. We therefore examined the interaction of tea components with the COVID-19 protein 5R84. Through molecular docking analysis, we identified three tea ingredients ((–)-epicatechin-3-o-gallate, D-(+)-cellobiose, and EGCG) that likely interact with the vital SARS-CoV-2 protein, 5R84, compared with the qualified 5R84 ligand WGS. According to the description in PubChem (https://pubchem.ncbi.nlm.nih.gov/compound/24802025#section=Household-Products), D-(+)-cellobiose is indeed insoluble in water and cannot be absorbed by the human body; thus, it is nearly impossible to inhibit SARS-CoV-2 through absorption from the gastrointestinal tract and into circulation. However, considering the droplet transmission and fecal-oral transmission of SARS-CoV-2, namely, that SARS-CoV-2 exists on the surfaces of the respiratory tract and digestive tract, D-(+)-cellobiose may directly interact with SARS-CoV-2 on the corresponding surfaces. However, the roles of (–)-epicatechin-3-o-gallate and D-(+)-cellobiose in COVID-19 should be studied further in cell and animal experiments.

In summary, our research systematically analyzed the active ingredients of tea, namely, (–)-epicatechin-3-o-gallate, D-(+)-cellobiose and EGCG, which have the potential to treat COVID-19 by suppressing the target genes and signaling pathways of COVID-19 and interacting with the vital SARS-CoV-2 protein. In addition, we validated the above results in macrophages. Our study analyzed the anti-COVID-19 effects of the active ingredients of tea and provided new ideas for the prevention and treatment of COVID-19.

## Data Availability Statement

The original contributions presented in the study are included in the article/Supplementary Materials, further inquiries can be directed to the corresponding author/s.

## Author Contributions

LW and QT: conception and design, collection and/or assembly of data, data analysis and interpretation, visualization, and manuscript writing and final approval of the manuscript. ZW and JS: collection and/or assembly of data. WY and LZ: collection and/or assembly of data. XY, LW, and YS: financial support, administrative support, provision of study material, supervision, data analysis and interpretation, visualization, manuscript writing, and final approval of the manuscript. All authors reviewed the manuscript. All authors have read and agreed to the published version of the manuscript.

## Funding

This work was supported by the National Key Research and Development Program of China (No: 2020YFC2005300), the Natural Science Youth Foundation of the Jiangsu Province (Grant BK20210074), the Introduction Program of high-level innovative and entrepreneurial talents in Jiangsu province, Wuxi first Double hundred Young and middle-aged Top-notch Medical and health talents Program (HB2020108), and Wuxi Health Commission scientific research project youth project (Q202059).

## Conflict of Interest

The authors declare that the research was conducted in the absence of any commercial or financial relationships that could be construed as a potential conflict of interest.

## Publisher's Note

All claims expressed in this article are solely those of the authors and do not necessarily represent those of their affiliated organizations, or those of the publisher, the editors and the reviewers. Any product that may be evaluated in this article, or claim that may be made by its manufacturer, is not guaranteed or endorsed by the publisher.
