# Supplementary material for: Tea Ingredients Have Anti-coronavirus Disease 2019 (COVID-19) Targets Based on Bioinformatics Analyses and Pharmacological Effects on LPS-Stimulated Macrophages
Source: Front Nutr. 2022 May 20;9:875765. doi: 10.3389/fnut.2022.875765 (PMC9163550; doi:10.3389/fnut.2022.875765)

Supplement Figure 1. The intersection of tea target genes with COVID19 signature genes. The target genes bar plot of each tea ingredients.

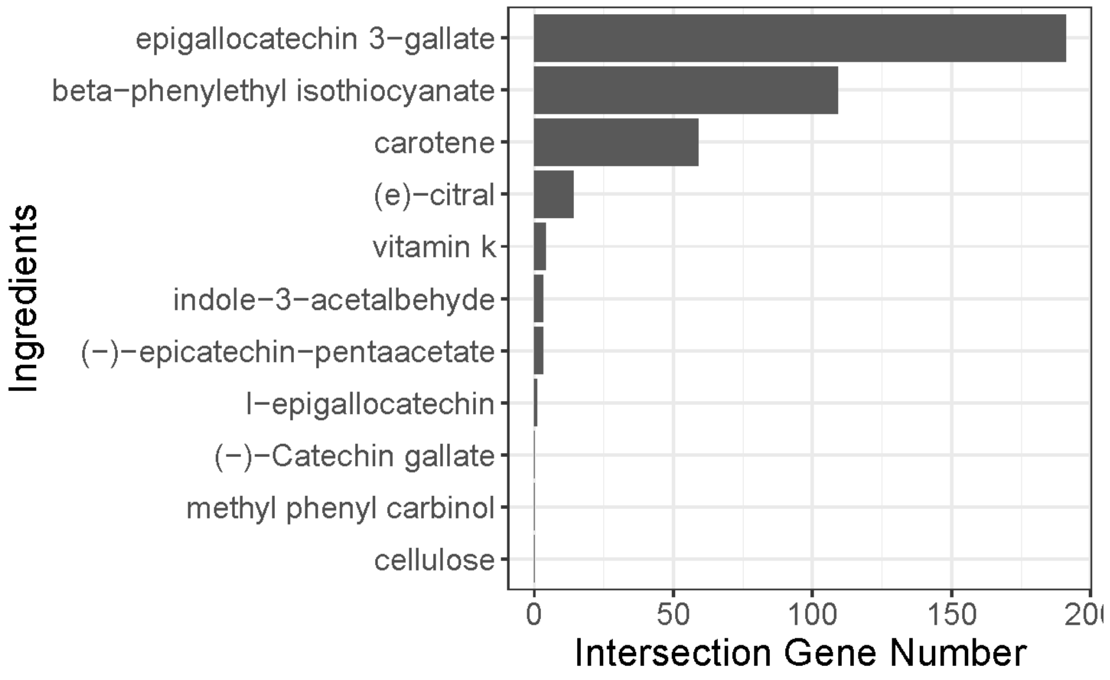

Supplement Figure 2. Tea COVID19 intersect gene enrichment.

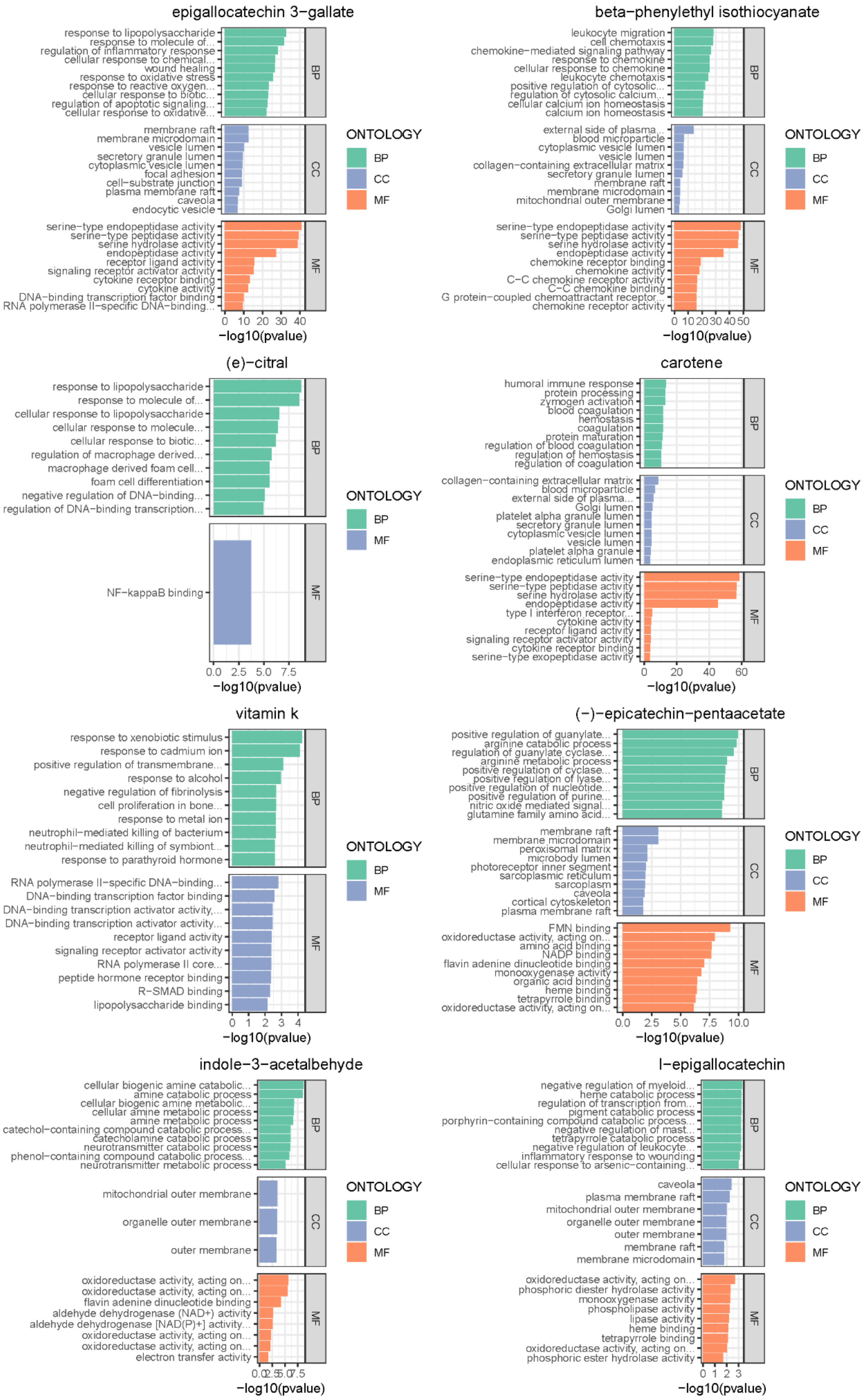

Supplement Figure 3. The inflammatory factor IL-17A secreted by macrophages and the mRNA expressions of *TLR4 and PI3K.* (A) The concentrations of IL-17A in RAW 264.7 cell supernatant after LPS (100 ng/ml) and EGCG (5-50 nM) treatment for 24 h were determined by ELISA kits (n=4). (B-C) The mRNA levels of *TLR4* and *PI3K* in the RAW 264.7 cells with LPS (100 ng/ml) and EGCG (5-50 nM) for 24 h were detected by q-PCR (n=3). *P < 0.1, **P < 0.01, ***P < 0.001.

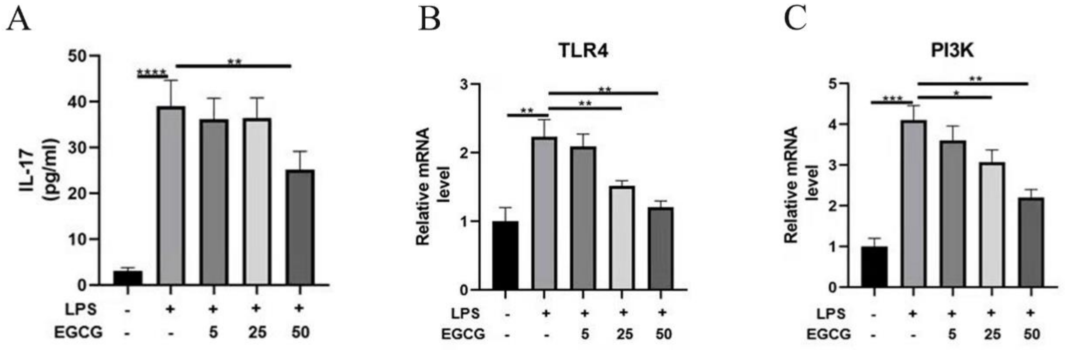

Supplement Figure 4. The molecular structures of tea ingredients.

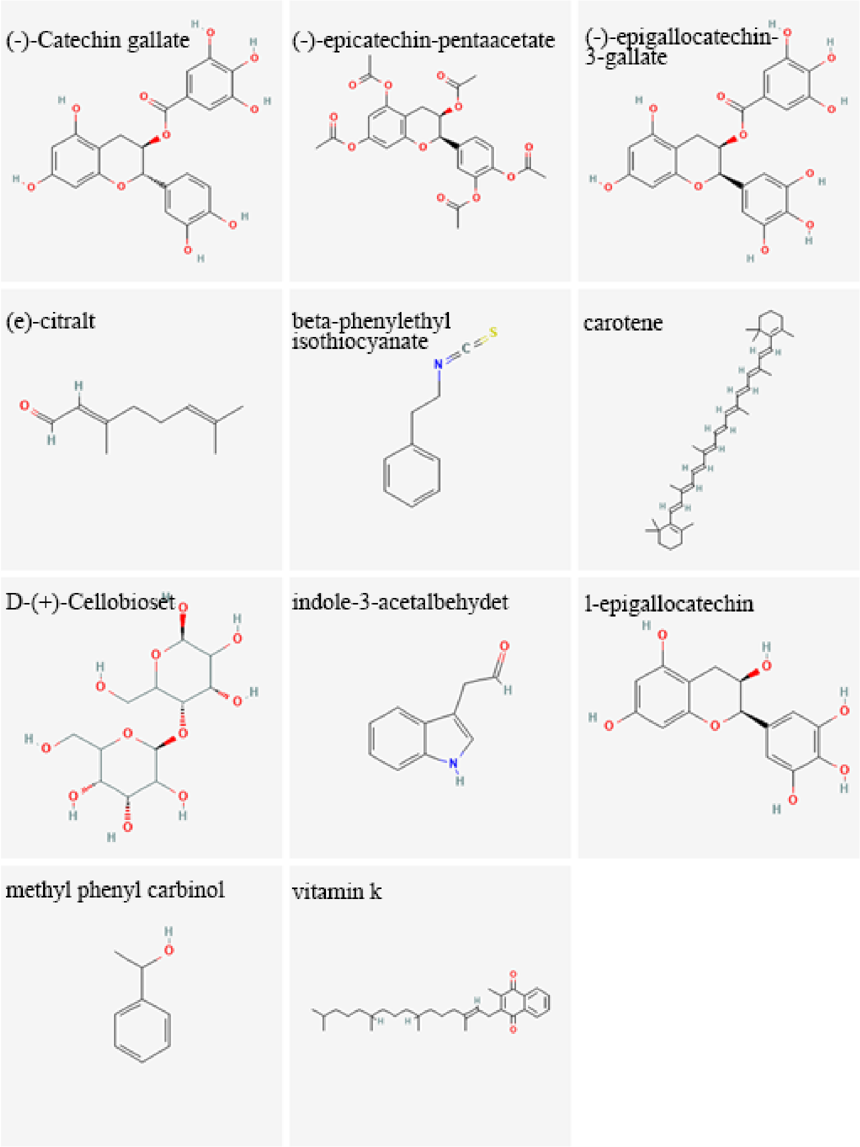

Supplement: Supplementary file 1 [file Image_1.PDF]
